# Integrating multiple machine learning algorithms for prognostic prediction of gastric cancer based on immune-related lncRNAs

**DOI:** 10.3389/fgene.2023.1106724

**Published:** 2023-04-04

**Authors:** Guoqi Li, Diwei Huo, Naifu Guo, Yi Li, Hongzhe Ma, Lei Liu, Hongbo Xie, Denan Zhang, Bo Qu, Xiujie Chen

**Affiliations:** ^1^ Department of Pharmacogenomics, College of Bioinformatics Science and Technology, Harbin Medical University, Harbin, China; ^2^ Department of General Surgery, The Fourth Affiliated Hospital of Harbin Medical University, Harbin, China; ^3^ Department of Biological Science, College of Biological Science and Technology, Harbin Normal University, Harbin, China; ^4^ Department of Gastroenterology, The Second Affiliated Hospital of Harbin Medical University, Harbin, China

**Keywords:** gastric cancer, machine learning, ILPM, prognosis, immunity

## Abstract

**Background:** Long non-coding RNAs (lncRNAs) play an important role in the immune regulation of gastric cancer (GC). However, the clinical application value of immune-related lncRNAs has not been fully developed. It is of great significance to overcome the challenges of prognostic prediction and classification of gastric cancer patients based on the current study.

**Methods:** In this study, the R package ImmLnc was used to obtain immune-related lncRNAs of The Cancer Genome Atlas Stomach Adenocarcinoma (TCGA-STAD) project, and univariate Cox regression analysis was performed to find prognostic immune-related lncRNAs. A total of 117 combinations based on 10 algorithms were integrated to determine the immune-related lncRNA prognostic model (ILPM). According to the ILPM, the least absolute shrinkage and selection operator (LASSO) regression was employed to find the major lncRNAs and develop the risk model. ssGSEA, CIBERSORT algorithm, the R package maftools, pRRophetic, and clusterProfiler were employed for measuring the proportion of immune cells among risk groups, genomic mutation difference, drug sensitivity analysis, and pathway enrichment score.

**Results:** A total of 321 immune-related lncRNAs were found, and there were 26 prognostic immune-related lncRNAs. According to the ILPM, 18 of 26 lncRNAs were selected and the risk score (RS) developed by the 18-lncRNA signature had good strength in the TCGA training set and Gene Expression Omnibus (GEO) validation datasets. Patients were divided into high- and low-risk groups according to the median RS, and the low-risk group had a better prognosis, tumor immune microenvironment, and tumor signature enrichment score and a higher metabolism, frequency of genomic mutations, proportion of immune cell infiltration, and antitumor drug resistance. Furthermore, 86 differentially expressed genes (DEGs) between high- and low-risk groups were mainly enriched in immune-related pathways.

**Conclusion:** The ILPM developed based on 26 prognostic immune-related lncRNAs can help in predicting the prognosis of patients suffering from gastric cancer. Precision medicine can be effectively carried out by dividing patients into high- and low-risk groups according to the RS.

## Introduction

Gastric cancer (GC) is a common tumor of the gastric mucosa. In 2020, GC ranked fifth and fourth in terms of cancer incidence and mortality worldwide, respectively (Sung et al., 2021). Nearly 90% of GC cases are caused by *Helicobacter pylori* (Plummer et al., 2015; Youn Nam et al., 2019). Currently, GC patients are mainly treated by surgery, chemotherapy, radiotherapy, etc., but the prognosis is still poor (Tan, 2019). The American Joint Committee on Cancer (AJCC) classification, as a traditional and commonly used tool to assess a patient’s condition based on the clinical stage, is often used as a reference for patient treatment demand. However, the AJCC has many limitations because it does not take into account molecular biological characteristics (Edge and Compton, 2010; Amin et al., 2017). Recently, immunotherapy represented by immune checkpoint inhibitors (ICIs) has emerged in GC (Bang et al., 2019a; Aoki et al., 2019; Bang et al., 2019b; Kulangara et al., 2019; Masuda et al., 2019; Roviello et al., 2019; Sunakawa et al., 2019). Programmed death-ligand 1 (PD-L1) expression, tumor mutational burden (TMB), neoantigen load (NAL), and mismatch repair deficiency (dMMR)/microsatellite instability-high (MSI-H), as the candidate biomarkers for ICI treatment, show good performance in small populations. Due to spatiotemporal heterogeneity, the accuracy of these approaches is very restrictive (Gibney et al., 2016; Cortes-Ciriano et al., 2017; Chan et al., 2019). Hence, identifying reliable biomarkers in GC is the goal of the current study.

GC is a disease with high heterogeneity between and within tumors. How to find the ideal biomarker is a question worthy of study. Therefore, a multigene panel, especially messenger RNAs (mRNAs) or microRNAs (miRNAs), based on microarray and RNA‐seq databases such as The Cancer Genome Atlas (TCGA) and Gene Expression Omnibus (GEO), was used to develop a prognostic gene signature by bioinformatics technology (Ren et al., 2020a; Wen et al., 2020; Zheng et al., 2021a). However, due to insufficient data utilization, single machine learning methods, and lack of sufficient verification and comparison, these studies’ results are difficult to apply in clinical settings (Bai et al., 2020; Ren et al., 2020b; Ji et al., 2020). Recently, studies have discovered that long non-coding RNAs (lncRNAs), with a length of more than 200 nucleotides and transcripts with no protein-coding capacity, can affect biological processes and the expression of multiple genes (Lin and Yang, 2018; Gugnoni and Ciarrocchi, 2019). CRNDE (Zhang et al., 2021), THAP7-AS1 (Liu et al., 2022), and UCA1 (Wang et al., 2019) can affect GC in different ways. Thus, the integration of lncRNA into the prognostic biomarker model of preclinical studies is quite necessary. Indeed, review studies show that lncRNAs are closely related to the tumorigenesis, metastasis, prognosis, and drug resistance of GC (Gu et al., 2015). The lncRNA-based model has been established in hepatocellular carcinoma (Sun et al., 2019), lung cancer (Zhou et al., 2019), and pancreatic cancer (Zhang et al., 2019). However, systematic studies on lncRNAs in GC are still few.

In this study, we obtained immune-related lncRNAs for gastric cancer based on the ImmLnc method and integrated multiple machine learning algorithms to construct a prognostic model for gastric cancer patients. The prognostic model outperformed other clinical features in both the training and validation sets. Patient subtypes based on prognostic risk scores differ in a variety of characteristics, including immune cell infiltration, drug sensitivity, and mutational characteristics. This study may help in improving the clinical prognosis and precise treatment of gastric cancer patients.

## Materials and methods

### Public data collection and processing

Here, we downloaded the RNA-seq raw read count, mutation data, and clinical data on stomach adenocarcinoma (STAD) from The Cancer Genome Atlas (TCGA) database. The read count was converted to transcripts per kilobase million (TPM) using the R package GenomicFeatures and further log-2 conversion. We kept the clinical information on age, grade, pathologic M stage, pathologic N stage, pathologic T stage, gender, and AJCC stage. Only samples with clinical information were retained. At the same time, microarray expression profile data and clinical information for two additional gastric cancer datasets GSE57303 and GSE62254 were all extracted from the Affymetrix GPL570 platform (Human Genome U133 Plus 2.0 Array) and were downloaded from the Gene Expression Omnibus (GEO) database. The raw data (.cel files) on GSE57303 and GSE62254 were processed by the robust multi-array average (RMA) algorithm *via* the R package Affy. We used the following steps for processing: 1) the samples without clinical data were removed; 2) the samples with no survival time, less than 0 days, or no survival status were removed; 3) the probe name was converted to the gene name; 4) the samples with multiple genes corresponding to one probe were removed; and 5) the mean value of multiple identical gene expression values was taken as the expression value of the gene. Following the gene annotations from the GENECODE (*Homo sapiens* GRCh38, releases V22), 17,450 protein-coding genes and 15,900 lncRNAs were included in the TCGA-STAD. Meanwhile, the gene symbols of GPL570 have annotated 17,912 protein-coding genes and 2415 lncRNAs.

### Identification of prognostic immune-related lncRNAs

ImmLnc was used in this study as an effective algorithm to identify immune-related lncRNAs based on immune-related pathways. The basic idea of the algorithm is as follows: 1) tumor purity was evaluated by the ESTIMATE algorithm; 2) partial correlation coefficients of mRNAs and lncRNAs were calculated by removing the influence of tumor purity; and 3) all mRNAs associated with a specific lncRNA were ranked according to the correlation coefficient, and the sorted gene list was analyzed for gene set enrichment analysis (GSEA). A default threshold of lncRES scores >0.995 and FDR <0.05 was used to screen immune-related lncRNAs. Based on immune-related lncRNAs, we carried out a univariate Cox regression analysis of immune-related lncRNAs with the coxph function of R package survival to find (*p* < 0.05) prognostic immune-related lncRNAs for prognosis.

Integration of multiple machine learning algorithms to construct an optimized immune-related lncRNA prognostic model (ILPM)

Here, we integrate 117 algorithm combinations from 10 machine learning algorithms to develop an ILPM with high accuracy and robust performance. The 10 algorithms are random survival forest (RSF), elastic network (Enet), Lasso, Ridge, stepwise Cox, CoxBoost, partial least squares regression for Cox (plsRcox), supervised principal components (SuperPC), generalized boosted regression modeling (GBM), and survival support vector machine (survival-SVM), respectively. After that, the 117 algorithm combinations were performed in the training dataset (TCGA-STAD) and two validation datasets (GSE57303 and GSE62254). In every model, Harrell’s concordance index (C-index) was calculated both in the training and validation datasets. We selected the model with the highest average C-index as the ILPM. The risk score of each gastric cancer sample was obtained by the ILPM, and the samples were divided into high- and low-risk groups according to the median.

### Estimation of the proportion of immune infiltrating cells

The CIBERSORT algorithm of the R package IOBR was used according to the expression profile of the TCGA-STAD dataset for measuring the proportion of immune infiltrating cells. The CIBERSORT algorithm is a method used to characterize the composition of cells based on the gene expression profile of complex tissues. We used the leukocyte characteristic gene matrix LM22 composed of 547 genes to differentiate 22 immune cell types, including myeloid subsets, plasma cells, naive and memory B cells, natural killer (NK) cells, and seven T-cell types. CIBERSORT, in combination with the LM22 characteristic matrix, was employed for estimating the proportion of 22 cell phenotypes in the sample. The sum of the proportions of all immune cell types in each sample was equal to 1. Meanwhile, the single-sample gene set enrichment analysis (ssGSEA) algorithm of the R package GSVA was employed for computing the 28 immune cells in the TCGA-STAD dataset.

### Genomic mutation and drug sensitivity analysis

A waterfall diagram was drawn using the R Package maftools to depict the variation distribution of genes between high- and low-risk groups. The R package pRRophetic and model gene expression data were employed to predict the sensitivity (IC_50_ value) of 138 medications in the Genomics of Drug Sensitivity in Cancer (GDSC) database. The IC_50_ value was used to determine how sensitive lung adenocarcinoma patients were to medication treatment. The Wilcoxon test examined the variations in IC_50_ values between high- and low-risk groups, and medications with significant variations between the two groups were discovered.

Biological function and pathway enrichment analysis of differentially expressed genes in high- and low-risk groups

Differentially expressed genes (DEGs) (*p*-value<0.05, log|FC|>1) between high- and low-risk groups were used for further study. Gene Ontology (GO) analysis is a common method for large-scale functional enrichment studies, including biological process (BP), molecular function (MF), and cellular component (CC). The Kyoto Encyclopedia of Genes and Genomes (KEGG) is a widely used database of information about genomes, biological pathways, diseases, and drugs. We used the R package clusterProfiler for GO annotation analysis of DEGs. The entry screening criteria included a *p*-value of <0.05, and the FDR value (q.vue) < 0.25 was considered statistically significant; *p*-values were corrected by the Benjamini–Hochberg (BH) method. Gene set enrichment analysis (GSEA) is a computational method to analyze whether a particular gene set is statistically different between two biological states. It is commonly used to estimate changes in the pathway and biological process activities in expression dataset samples. In this study, the clusterProfiler package was used for the enrichment analysis of DEGs. The parameters used in the GSEA were as follows: the number of genes contained in each gene set was at least 10, and the number of genes contained in each gene set was at most 500. The *p*-value correction method used was the Benjamini–Hochberg (BH) method. Downloaded from MSigDB database. The “c2. Cp. Kegg. 7.5.1. Symbols. gmt” and “c5. Go. 7.5.1. Symbols. gmt” were downloaded from MSigDB database as a reference gene set.

Gene set variation analysis (GSVA) is a non-parametric unsupervised analytical method, which is mainly evaluated by transforming the expression matrix of genes among different samples into the expression matrix of genes among samples. Transcriptome gene set enrichment results were analyzed to assess whether different metabolic pathways are enriched between different samples. To study the biological process variation between high- and low-risk groups, we used the R package “GSVA” to conduct gene set variation analysis based on the TCGA-STAD gene expression profile dataset. The reference gene set “h.all.v2022.1. Hs.symbols.gmt” which downloaded from MSigDB database was used to compute dataset every sample enrichment of reference genes in each set of scores, GSVA scores between high- and low-risk group, and *t*-test *p*-value is less than 0.05 is considered a significant difference.

### Statistical analysis

The Wilcoxon test was used in significance labeling for comparing the differences between the two groups of samples, and the Kruskal–Wallis test helped in comparing the differences between multiple groups of samples. Here, ns indicates *p* ≤ 0.05, * indicates *p* ≤ 0.05, ** indicates *p* ≤ 0.01, *** indicates *p* ≤ 0.001, and ****indicates *p* ≤ 0.0001. Among them, *p* < 0.05 was significant, and the difference was statistically significant. The receiver operating characteristic curve (ROC) was implemented *via* the pROC package. The time-dependent area under the ROC curve (AUC) for survival variables was proved by the timeROC package. The Kaplan–Meier survival analysis and univariate and multivariate analyses with Cox proportional hazard regression for overall survival (OS) and disease-free survival (DFS) were carried out using R packages survivalROC and survminer. All the statistical analyses were performed in R 4.1.1 software.

## Results

### Obtaining immune-related lncRNAs from the ImmLnc algorithm

**FIGURE 1:**
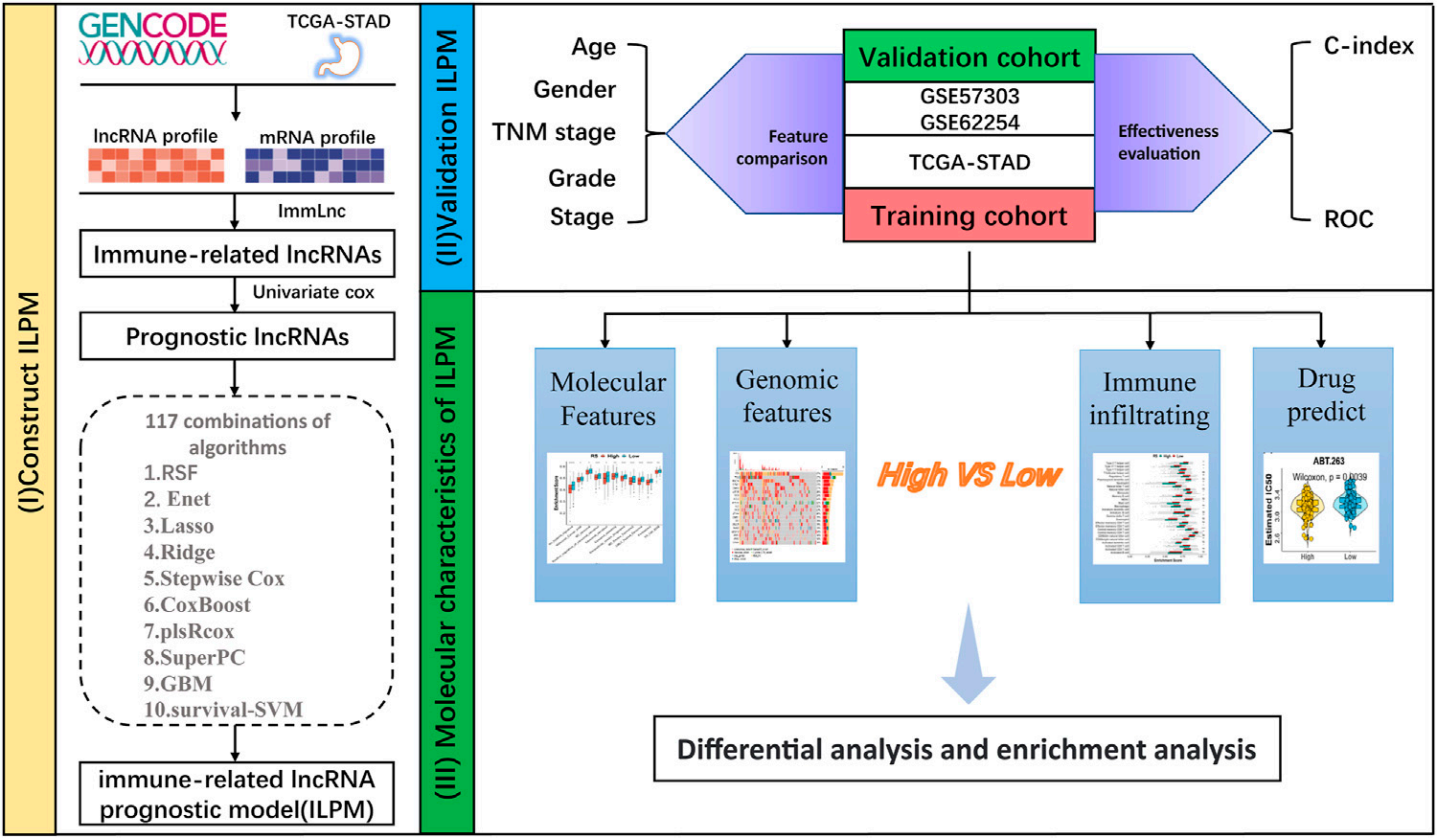
Workflow of this study.

**FIGURE 2:**
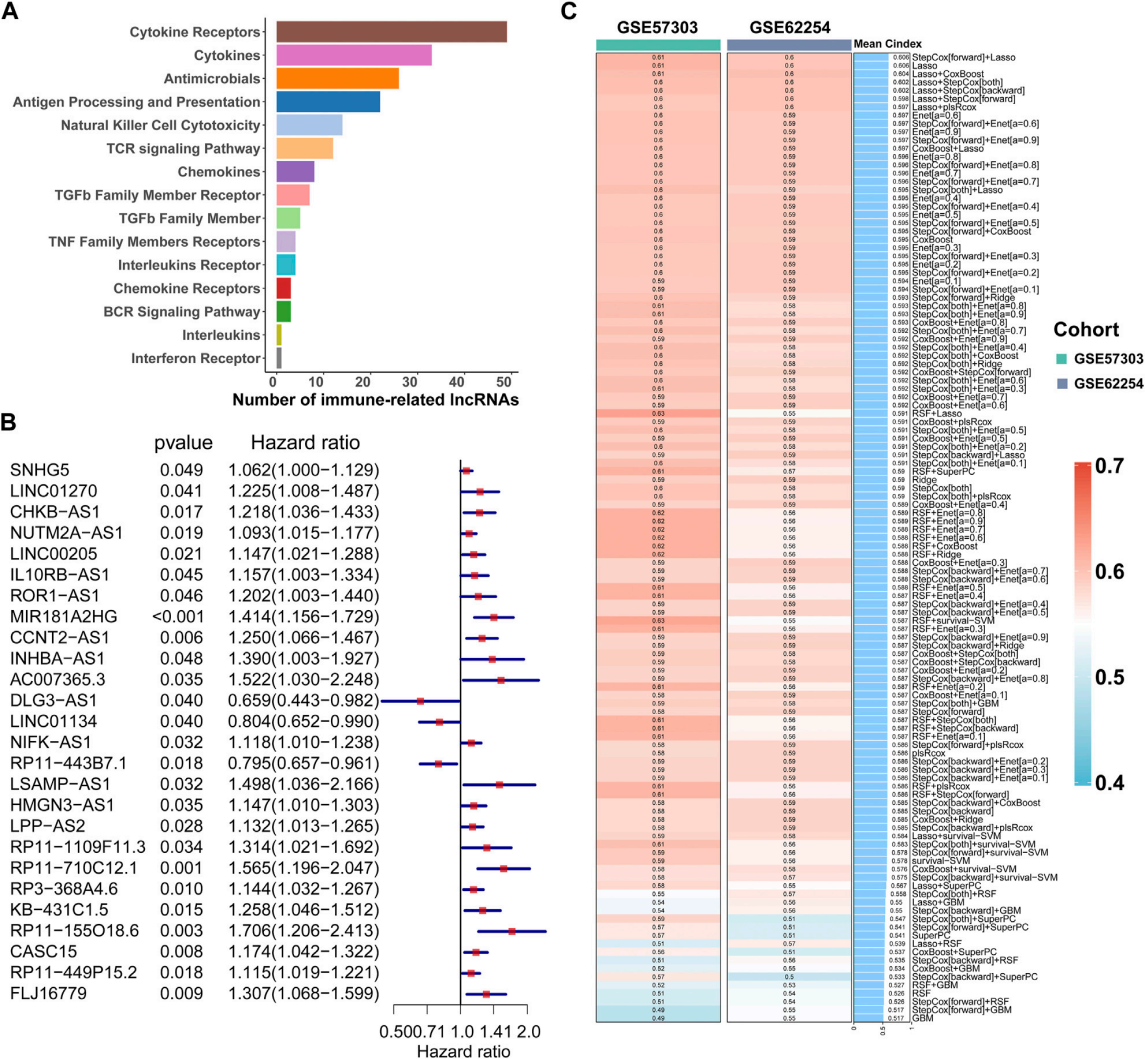
Calculation of the C-index of 117 integrated machine learning algorithms. **(A)** ImmLnc identified a total of 321 lncRNAs significantly associated with immune‐related pathways. **(B)** Univariate Cox regression analysis of OS obtained 26 prognostic immune-related lncRNAs in the TCGA-STAD dataset (n = 296). Data are presented as a hazard ratio (HR) ± 95% confidence interval [CI]. **(C)** C-index of 117 kinds of prediction models was calculated across two validation datasets.

The workflow of this study is shown in Figure 1. According to the expression profile data on mRNAs and lncRNAs, ImmLnc can obtain immune-related lncRNAs according to immune-related pathways. The assumption is that, if a lncRNA plays an important role in immune regulation, its related genes will be enriched in the upper or lower part of immune-related pathways. In this study, 321 immune-related lncRNAs were identified using the ImmLnc algorithm, and these lncRNAs are significantly correlated with “Cytokine Receptors,” “Cytokines,” “Antimicrobials,” and “Antigen Processing and Presentation” pathways (Figure 2A).

### Integrating machine learning algorithms to construct the ILPM

**FIGURE 3:**
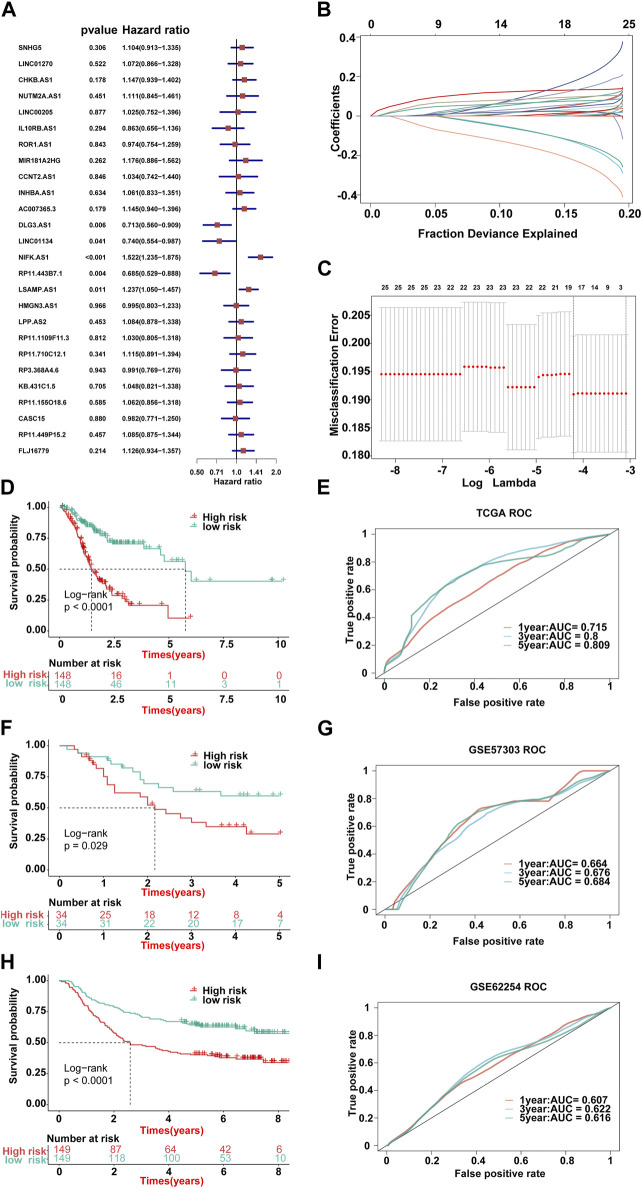
Lasso result diagram of TCGA training set and prognostic efficacy of the model across all datasets. **(A)** Multivariable Cox regression analysis (direction = forward) of OS in the TCGA-STAD dataset (n = 296), with no culling of variables. Data are presented as a hazard ratio (HR) ± 95% confidence interval [CI]. **(B)** Changing track of the Lasso regression independent variable; the abscissa represented the logarithm of the independent variable lambda, and the ordinate represented the coefficient of the independent variable. **(C)** Confidence interval under each lambda of Lasso. **(D–I)** Kaplan–Meier curve of OS according to the ILPM in the TCGA-STAD (n = 296, log-rank test: *p* <0.001): **(D)** GSE57303 (n = 68, log-rank test: *p* = = 0.029); **(F)** GSE62254 (n = 298, log-rank test: *p* <0.001); and **(H)** corresponding ROC curves for predicting OS at 1, 3, and 5 years in TCGA-STAD **(E)**, GSE57303 **(G)**, and GSE62254 **(I)**.

First of all, according to the expression profile data on the 321 immune-related lncRNAs from TCGA-STAD, we identified 26 prognostic lncRNAs by the univariate Cox regression analysis (Figure 2B). Then, we added these 26 lncRNAs into 117 integrated machine learning algorithms to develop the ILPM. The C-index was calculated through the leave-one-out cross-validation (LOOCV) framework both in the TCGA training dataset and GEO validation datasets (Figure 1C). It is worth noting that stepwise Cox (direction = forward) and Lasso algorithm combined with the Lasso algorithm has the highest average C-index (0.606) (Figure 2C). The average C-index calculated by Lasso is the highest because the stepwise Cox (direction = forward) algorithm does not eliminate lncRNAs, which can be seen in Figure 3A. In the Lasso algorithm, after removing duplicated genes with Lasso linear regression, the ILPM was built, and 18 prognostic immune-related lncRNAs (SNHG5, LINC01270, CHKB. AS1, NUTM2A.AS1, MIR181A2HG, CCNT2.AS1, DLG3.AS1, LINC01134, NIFK.AS1, RP11.443B7.1, LSAMP.AS1, HMGN3.AS1, LPP.AS2, RP11.710C12.1, RP11.155O18.6, CASC15, RP11.449P15.2, and FLJ16779) were discovered. Figures 3B, C show the Lasso results and the weight coefficient of each lncRNA, as shown in Table 1. Among the 18 lncRNAs, the single-factor Cox regression *p*-value of MIR181A2HG is the smallest, indicating that it has the best prediction effect. The *MIR181A2HG* gene is a member of the *MIR181A2* host gene, which is located in the nucleus and expressed in most tissues of the human body (Fagerberg et al., 2014). As an immune-related lncRNA, the widespread expression of the *MIR181A2HG* gene in human tissues may indicate that mir181A2Hg plays a very important role in immune regulation. However, the current research on *MIR181A2HG* is limited (Bonaldo et al., 1996; Lytle et al., 2004; Oshikawa et al., 2011; Wang et al., 2021), so *MIR181A2HG* has high research values and is worth further analysis in subsequent studies. Then, a risk score (RS) for each patient was calculated based on the Lasso algorithm for 18 lncRNA coefficients. All patients were divided into high- and low-risk groups according to the optimal cut-off value defined in the R package survminer. Afterward, we assessed the impact of the model scores developed by the 18 lncRNA signatures on the overall survival of the training dataset. The outcomes are illustrated in Figure 3D. Samples in the high-risk group had a worse prognosis, and the KM curve of the high-risk group was *p* < 0.0001, showing that there were major variations in the prognosis of the two groups. According to the developed ILPM, the ROC curve of the prognostic signature was drawn, as illustrated in Figure 3E. The AUC values of 1/3/5 years were 0.715/0.8/0.809, respectively, showing good prediction efficiency of the model score. The capacity of RS to predict overall survival was then tested using the validation datasets. In the validation datasets, the samples were sorted into high- and low-risk groups using the same technique as the TCGA training dataset. The prognosis of the high-risk group was worse, as indicated in Figures 3F, H, and there were substantial disparities in the prognosis of the high- and low-risk groups. As shown in Figures 3G, I, the AUC of 1/3/5 years in the GSE57303 validation dataset was 0.664/0.676/0.684, and the AUC of 1/3/5 years in the GSE62254 validation dataset was 0.607/0.622/0.616. The aforementioned results indicate that the ILPM had a strong and stable efficiency for survival prediction in the TCGA training dataset and GEO validation datasets.

**TABLE 1:** Weight coefficients of 18 lncRNAs in Lasso.

LncRNA	Weight coefficient
SNHG5	0.046406
LINC01270	0.002013
CHKB.AS1	0.082716
NUTM2A.AS1	0.042071
MIR181A2HG	0.130342
CCNT2.AS1	0.007713
DLG3.AS1	−0.1127
LINC01134	−0.11123
NIFK.AS1	0.153615
RP11.443B7.1	-0.20088
LSAMP.AS1	0.116342
HMGN3.AS1	0.029911
LPP.AS2	0.013745
RP11.710C12.1	0.093786
RP11.155O18.6	0.074202
CASC15	0.024295
RP11.449P15.2	0.003993
FLJ16779	0.054282

### Checking the strength of the ILPM

**FIGURE 4:**
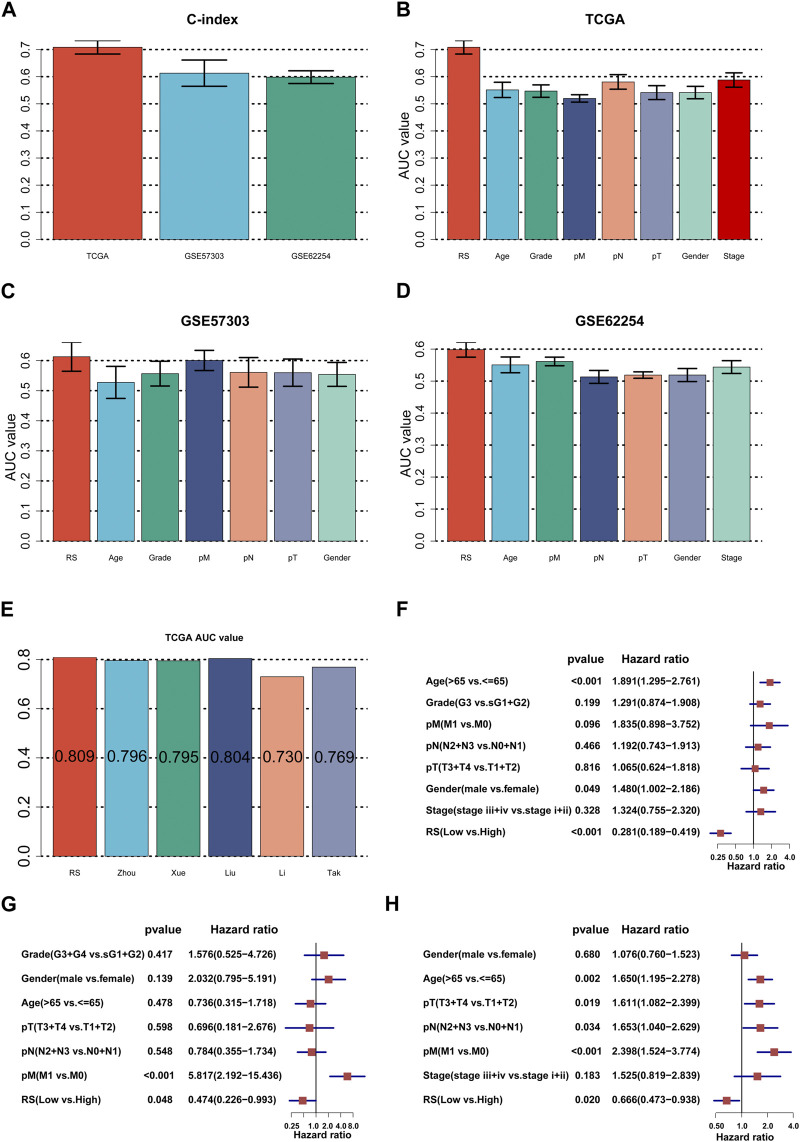
Assessment of the ILPM. **(A)** C-index of the ILPM in all datasets. **(B–D)** Performance of the ILPM compared with other clinical variables in predicting prognosis in TCGA-STAD (n = 296) **(B)**, GSE57303 (n = 68) **(C)**, and GSE62254 (n = 298) **(D)**. **(E)** AUC value of the ILPM and five published signatures in TCGA-STAD. **(F–H)** Multivariate Cox regression analysis of RS and other clinical variables in TCGA-STAD **(F)**, GSE57303 **(G)**, and GSE62254 **(H)**. Data are presented as the hazard ratio (HR) ± 95% confidence interval [CI].

**FIGURE 5:**
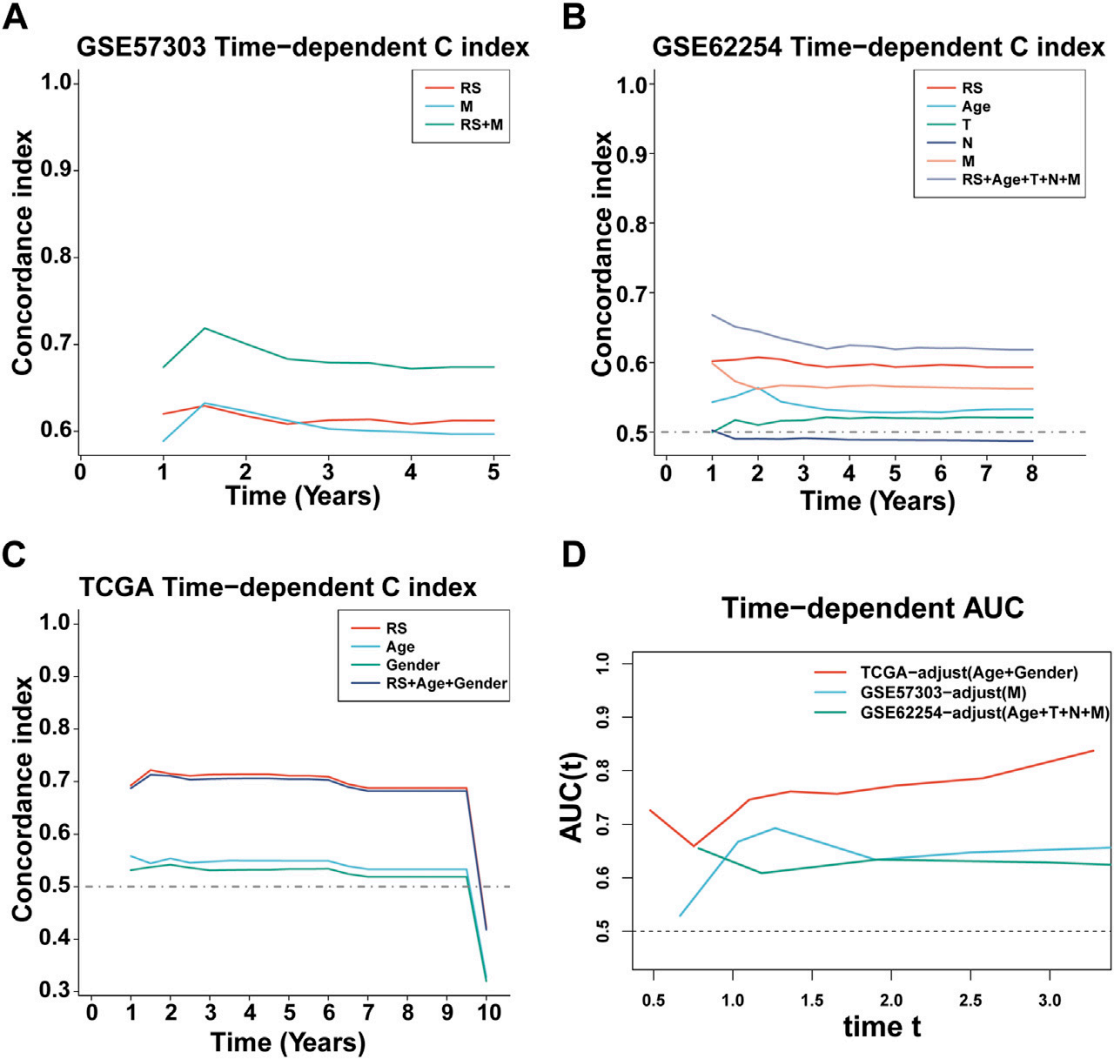
C-index and AUC assessment of RS and clinical variables. **(A–C)** C-index analysis of RS and clinical variables in TCGA-STAD (n = 296) **(A)**, GSE57303 (n = 68) **(B)**, and GSE62254 (n = 298) **(C)**. **(D)** Time-dependent ROC analysis for predicting OS in all datasets.

First, we calculated the C-index [95% confidence interval], which was 0.708 [0.683–0.733] in TCGA-STAD, 0.613 [0.565–0.661] in GSE57303, and 0.598 [0.575–0.622] in GSE62254 (Figure 4A). Furthermore, we also calculated the C-index of other clinical variables. In evidence, RS had distinctly superior accuracy compared to the other variables including age, grade, pathologic M stage, pathologic N stage, pathologic T stage, gender, and AJCC stage in TCGA-STAD (Figure 4B); age, grade, pathologic M stage, pathologic N stage, pathologic T stage, and gender in GSE57303 (Figure 4C); and age, pathologic M stage, pathologic N stage, pathologic T stage, gender, and AJCC stage in GSE62254 (Figure 4D). In addition, we compared the ILPM with five other models, such as those by Cai et al. (2019), Zheng et al. (2021b), Lei et al. (2022a), Li et al. (2021), and Takeno et al. (2010), for predicting the prognosis of patients with gastric cancer and found that the ILPM had the highest efficacy (Figure 4E). Second, independent prognostic factors were chosen for multivariate Cox regression analysis and discovered that RS, age, and gender in TCGA-STAD (Figure 4F); RS and pathologic M stage in GSE57303 (Figure 4G); and RS, age, pathologic M stage, pathologic N stage, and pathologic T stage in GSE62254 (Figure 4H) were independent prognostic factors. An interesting idea is to combine the ILPM with independent prognostic factors to further elevate clinical application. Therefore, we combined the ILPM with other clinical features (multivariate Cox regression analysis: *p* < 0.05) to predict patient prognosis and found that the combined model could better predict the prognosis (Figures 5A, B), except that there was a little difference in TCGA-STAD (Figure 5C). These results explain that the combination of RS and other clinical variables can significantly improve the ability to predict the prognosis of patients. Finally, we intended to check the strength of the ILPM by adjusting the interference of other variables because clinical variables significantly associated with prognosis can affect the predictive power of the ILPM. Here, we generated three datasets of time-dependent ROC analysis for predicting OS after adjusting age and gender in TCGA-STAD; pathologic M stage in GSE57303; and age, pathologic M stage, pathologic N stage, and pathologic T stage in GSE62254. The AUC value of time-dependent ROC analysis was high in all datasets (Figure 5D). In conclusion, the ILPM can effectively predict the prognosis of patients in various situations.

### The difference in the molecular and genomic features

**FIGURE 6:**
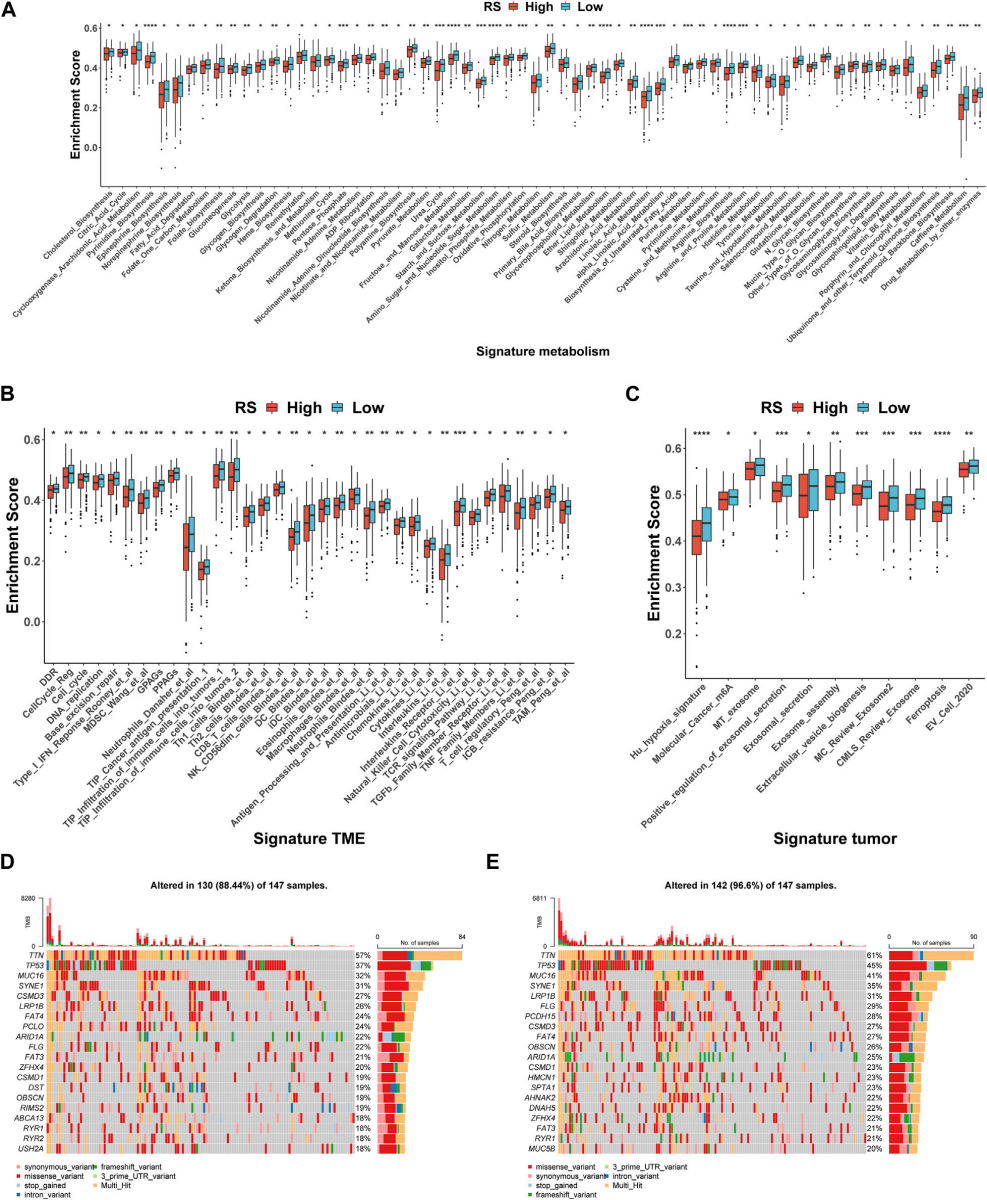
Molecular and genomic features between high- and low-risk groups in TCGA-STAD. **(A–C)** Box plot of metabolism signatures **(A)**, TME signatures **(B)**, and tumor signatures **(C)** in high- and low-risk groups. *p < 0.05; **p < 0.01; ***p < 0.001; ****p < 0.0001. **(D–E)** SNV waterfall of top 20 (mutation frequency) genes in the high-risk group (n = 147) **(D)** and low-risk group (n = 147) **(E)**.

In the occurrence and development of cancer, a variety of molecular characteristics will be accompanied by changes. Here, we mainly study the signatures of the tumor immune microenvironment (TIME), tumor metabolism, and the tumor itself, which were collected by the R package IOBR. Of the 114 metabolism signatures collected, 64 (Figure 6A) were significantly different between the high- and low-risk groups (35 of 119 TME signatures (Figure 6B) and 11 of 16 tumor signatures (Figure 6C)). These findings suggest that there are multiple characteristic differences between the high- and low-risk groups of patients according to our RS. Subsequently, the mutation landscape of the high- (Figure 6D) and low-risk (Figure 6E) groups of TCGA-STAD in the genome was displayed. As is known, a gene mutation might either promote or cause cancer, or it could coordinate and drive cancer’s malignant progression. The research and development of tumor-targeted medications and innovative tumor therapies relied heavily on knowledge of genome-level mutation. As demonstrated in Figures 6D, E, the distribution of somatic variation in each sample between high- and low-risk groups, in which only the top 20 genes with the highest mutation frequency were selected to draw a waterfall diagram, was different.

### The difference in the proportion of immune infiltrating cells

**FIGURE 7:**
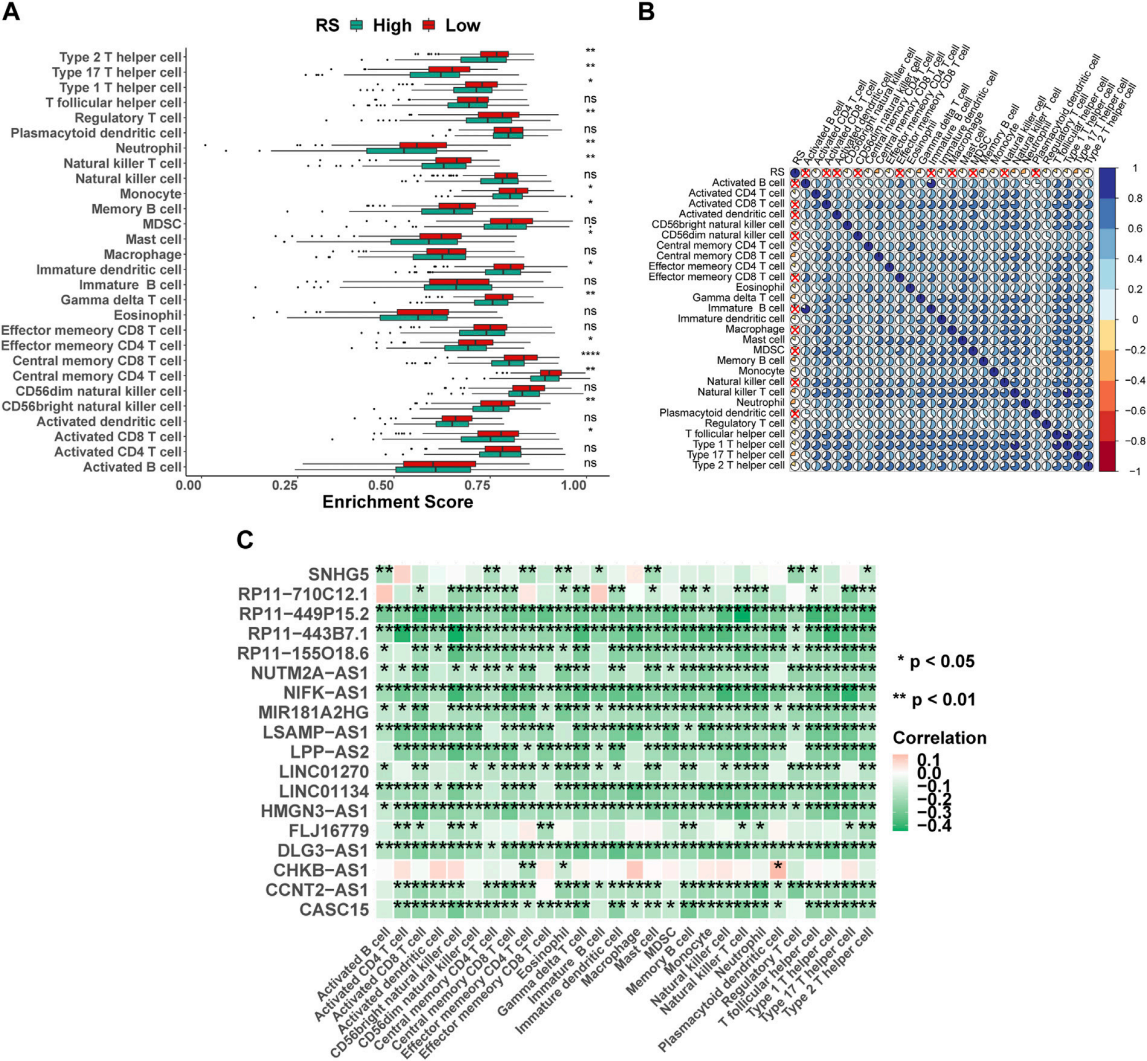
Proportion of infiltration of 28 immune cells was evaluated based on ssGSEA. **(A)** Box plot of the proportion of immune infiltrating cells in high- and low-risk groups. Green represents the high-risk group, and red represents the low-risk group. **(B)** RS and 28 immune cell correlation heat map. The cross marks represent a non-significant correlation, where blue is positive and red is negative. **(C)** 18 lncRNAs and 28 immune cell correlation heat map, where pink represents positive and green represents negative. *p < 0.05; **p < 0.01; ***p < 0.001; ****p < 0.0001.

Immune cells were the key categories of non-tumor components in the tumor microenvironment, and they had been postulated to be useful for tumor diagnosis and evaluating the prognosis. In this study, according to the signature genes of 28 immune cells, we calculated the percentage of infiltrated immune cells based on ssGSEA. Between the high- and low-risk groups, 16 of the 28 immune cells were significant differences (Figure 7A), for example, Type 2 T helper cells, Type 17 T helper cells, and Type 1 T helper cells. At the same time, the results of Spearman’s correlation analysis of RS and immune cell contents showed that the content of most immune cells was significantly correlated with RS (Figure 7B). Furthermore, the 18 lncRNAs that were used to construct the ILPM were also significantly correlated with most immune cell contents (Figure 7C), indicating that these lncRNAs played an important role in regulating the content of immune cells. To more comprehensively assess the degree of immune cell infiltration, we used the CIBERSORT algorithm to evaluate the proportion of immune cell infiltration. Although the results were significantly different from ssGSEA, the difference between natural killer cell content in the high- and low-risk groups and the significant correlation between the 18 lncRNAs and the content of immune cells were consistent (Supplementary Figure S4).

### Predicting the sensitivity of drugs between high- and low-risk groups

**FIGURE 8:**
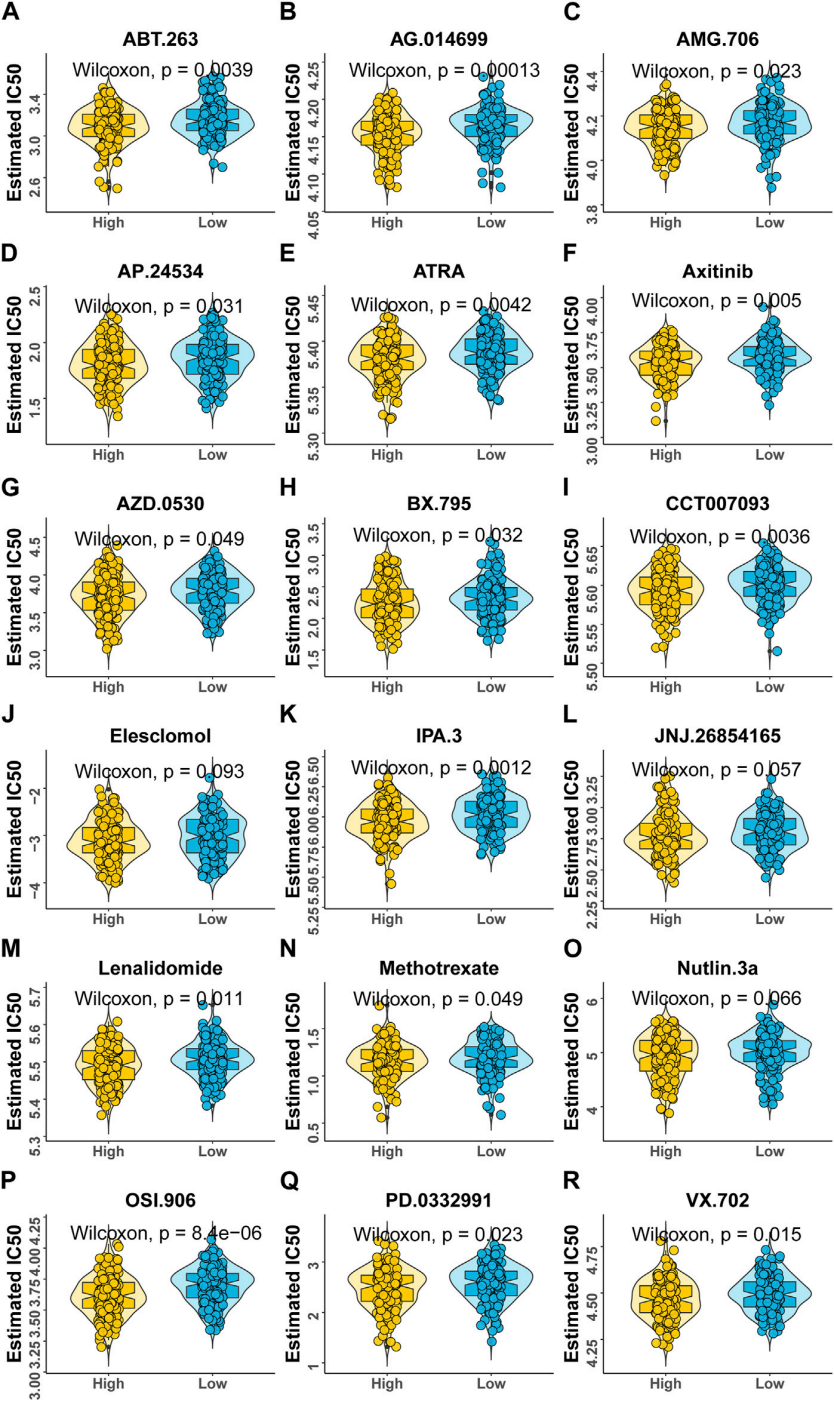
Variations in drug sensitivity between model groups. **(A–R)** IC_50_ box diagram of 18 drugs with the significant difference in drug sensitivity in the high- and low-risk groups, respectively, in which yellow represents the high-risk group and blue represents the low-risk group.

According to the expression profile data from TCGA-STAD, the sensitivity IC_50_ value of 138 drugs in the Genomics of Drug Sensitivity in Cancer (GDSC) database was predicted, of which 18 drugs had significant differences between high-risk and low-risk groups, which is displayed in Figure 8. These drugs work in different ways to suppress tumor growth, and it can be seen that patients in the high-risk group are more sensitive to these drugs, indicating that the treatment of patients in the high-risk group will be better. The functions and targets of 18 drugs are listed in Table 2.

**TABLE 2:** Function and target of 18 drugs.

Drug	Function	Target
ABT-263	Targets the Bcl-2 family of proteins, the major negative regulators of apoptosis	BCL2, BCL2L2, and BAD
AG.014699	Anticancer drug and poly (ADP-ribose) polymerase (PARP) inhibitor	PARP1, PARP2, and PARP3
AMG.706	Receptor tyrosine kinase inhibitors	VEGFR1, VEGFR2, VEGFR3, Kit, PDGF, and Ret
AP.24534	Multi-target kinase inhibitor	ABL1, BCR, KIT, RET, TEK, FLT3, FGFR1, FGFR2, FGFR3, FGFR4, LCK, SRC, LYN, KDR, PDGFRA, CYP3A4, CYP2C8, CYP2D6, CYP3A5, ABCB1, and ABCG2
ATRA	Naturally occurring derivative of vitamin A (retinol)	RXRB, RXRG, RARG, ALDH1A1, GPRC5A, GPRC5A, ALDH1A2, RARRES1, RARA, RARB, LCN1, OBP2A, RBP4, PDK4, RXRA, CYP26A1, CYP26B1, CYP26C1, and HPGDS
Axitinib	Selectively blocks the tyrosine kinase receptors VEGFR-1 (vascular endothelial growth factor receptor), VEGFR-2, and VEGFR-3	FLT1, KDR, and FLT4
AZD.0530	Src inhibitor	Src, FAK, p-FAK, and pSTAT-3
BX.795	Selective inhibitors of PDK1	PDK1, TBK1, and IKKε
CCT007093	Effective protein phosphatase 1D (PPM1D Wip1) inhibitor	WIP1
Elesclomol	Acts through a novel mechanism of action	FDX1
IPA.3	Selective non-ATP competitive PAK1 inhibitor	PAK1
JNJ.26854165	Acts as an HDM2 ubiquitin ligase antagonist and also induces early apoptosis in p53 wild-type cells and inhibits cellular proliferation, followed by delayed apoptosis in the absence of functional p53	HDM2 and Mdm2
Lenalidomide	Exerts immunomodulating effects by altering cytokine production, regulating T- cell co-stimulation, and enhancing the NK cell-mediated cytotoxicity	CRBN, TNFSF11, and CDH5
		PTGS2
Methotrexate	Enters tissues and is converted to a methotrexate polyglutamate by folylpolyglutamate	TYMS, ATIC, and DHFR
Nutlin.3a	Can inhibit the interaction between MDM2-p53 and stabilize p53 protein, and induce autophagy and apoptosis	MDM2-p53
OSI.906	IGF-1R stimulates proliferation, enables oncogenic transformation, and suppresses apoptosis	INSR and IGF1R
PD.0332991	Cyclin-dependent kinase 4/6 (CDK4/6) inhibitor 1 that acts by binding to the ATP pocket	CDK4 and CDK6
VX.702	Highly selective inhibitor of p38α MAPK, 14-fold higher potency against p38α *versus* p38β	p38α MAPK

## Identification of DEGs

**FIGURE 9:**
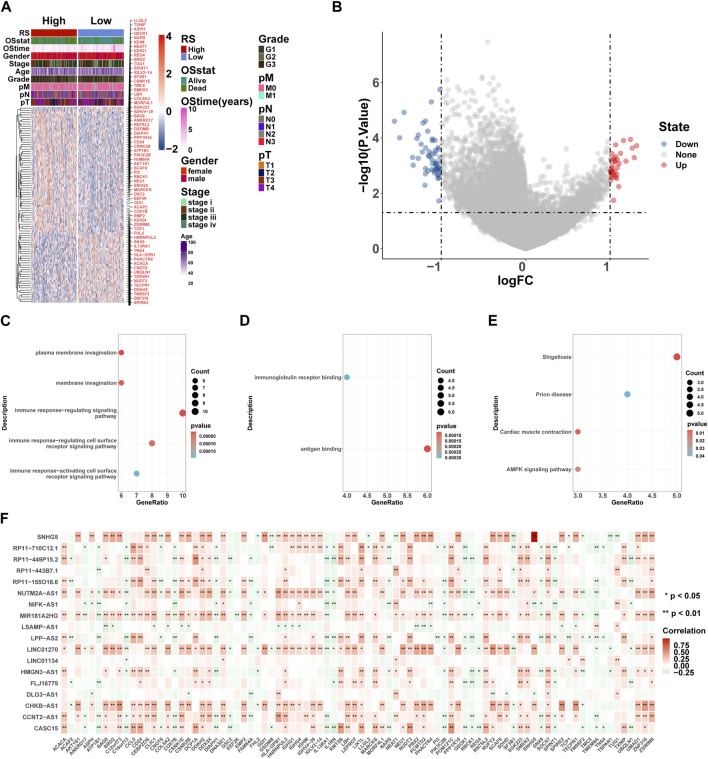
GO and KEGG enrichment analyses of DEGs. **(A)** Heatmaps of DEGs based on RS. **(B)** Volcano plot of DEGs based on RS. **(C–E)** Gene ontology (GO) and Kyoto Encyclopedia of Genes and Genomes (KEGG) pathway enrichment analysis of DEGs, including biological process (BP) **(C)**, molecular function (MF) **(D)**, and KEGG pathway **(E)**. Count: number of genes related to the enriched GO or KEGG pathway. The color of the bar denotes the *p*-value. **(F)** 18 lncRNA and 86 DEG correlation heat map, where red represents positive and green represents negative. *p < 0.05; **p < 0.01; ***p < 0.001; ****p < 0.0001.

Here, we performed differential expression analysis to identify differentially expressed genes between high- and low-risk groups. For comparison, according to RS, there were 53 upregulated and 33 downregulated genes in the high-risk group. Heatmaps showed 86 DEGs between high- and low-risk groups (Figure 9A), and the volcano plot showed the differential expression of genes at a set threshold in Figure 9B; see Table 3 for details of DEGs. To explore the potential molecular mechanism of DEGs, functional enrichment analysis was conducted for these genes. As shown in Figures 9C-E, the DEGs were mainly enriched in the external side of the immune response-regulating signaling pathway, immune response-regulating cell surface receptor signaling pathway, immune response-activating cell surface receptor signaling pathway, immunoglobulin receptor binding (GO terms), and the AMPK signaling pathway (KEGG pathway). These pathways are all related to immunity, which further indicates that patients in high- and low-risk groups have significant differences in immune-related pathways. To understand the regulatory relationship between these 18 lncRNAs and 86 DEGs, the results showed a significant correlation between the 18 lncRNAs and 86 DEGs (Figure 9F).

**TABLE 3:** DEGs between high‐ and low-risk groups.

Symbol	logFC	*p-*value	Adj. *p-*value
C12orf73	−1.01536	1.79E–06	0.008568
RUFY2	−1.11769	5.01E–06	0.011618
POM121C	−1.44039	1.20E–05	0.017033
UMAD1	−1.18662	2.46E–05	0.022458
DDX3X	−1.55441	2.50E–05	0.022458
LIX1L	−1.21549	2.91E–05	0.023438
SOCS7	−1.16048	5.71E–05	0.031613
KMT2B	−1.27127	7.84E–05	0.036373
CLIC1	−1.4925	9.92E–05	0.039587
CCL5	−1.28863	0.000105	0.040205
PCMTD2	−1.10561	0.000109	0.040559
TMEM8A	1.241609	0.000113	0.041219
DNASE1	−1.07316	0.000122	0.042165
IL1RN	1.158774	0.000124	0.042595
SPIRE2	−1.10699	0.000125	0.042767
CEBPZOS	−1.2383	0.000168	0.047219
C16orf13	1.065904	0.000178	0.048337
TM9SF2	1.313672	0.000191	0.050064
NUDT3	−1.03868	0.000226	0.053636
DDAH2	−1.18357	0.000229	0.053683
TSPAN1	1.262579	0.000234	0.053687
TECPR1	−1.0307	0.000254	0.05526
ZNF316	−1.07209	0.000285	0.058966
PHACTR4	−1.14247	0.000318	0.061099
ACACA	−1.22473	0.000323	0.061477
UBQLN1	1.123594	0.000361	0.063433
CNOT6	1.020255	0.000364	0.063433
PBX2	−1.07178	0.000369	0.063857
DCP1A	−1.1095	0.000384	0.064826
LEPROT	−1.15353	0.000439	0.068862
SDHD	−1.13977	0.000467	0.070208
SNX9	1.064844	0.000475	0.070391
HLA-DPB1	−1.33952	0.000507	0.071817
NEU1	−1.01464	0.000517	0.071856
PI3	1.286015	0.000519	0.071856
MARCKS	−1.27209	0.000549	0.07284
EEF2K	1.041823	0.00056	0.07295
GDI1	−1.11202	0.000601	0.075319
DSC2	1.084682	0.000602	0.075319
ACAP2	1.010065	0.000603	0.075319
ATP1B1	1.187554	0.000696	0.079982
PIK3C2B	1.060851	0.000714	0.079994
COX7B	1.047242	0.000718	0.079994
CSNK2B	−1.06363	0.00073	0.080369
SCAF8	−1.01112	0.000816	0.085172
SNHG5	−1.06858	0.000831	0.085172
FAM84A	1.107621	0.000846	0.085749
PPP1R10	−1.10321	0.000853	0.085749
AKT1S1	−1.0655	0.000931	0.087777
CD24	−1.33256	0.000968	0.090675
RBCK1	1.023269	0.001036	0.094091
ZSWIM8	−1.05491	0.001107	0.096042
FHL2	1.087134	0.001171	0.097449
HNRNPUL2	−1.03214	0.001176	0.097449
TCP1	1.018362	0.001261	0.100135
IGHG4	−1.18658	0.001275	0.100181
LBH	−1.03128	0.001282	0.100181
SMEK2	−1.06439	0.001364	0.103319
COL5A2	−1.11299	0.001392	0.104043
MORF4L1	−1.05974	0.00144	0.104559
SGK223	−1.01042	0.001447	0.10473
ANKRD17	1.023024	0.001495	0.106146
IGHV4-39	−1.01903	0.001539	0.107842
BAG6	−1.08612	0.001549	0.108232
RN7SL2	−1.1568	0.001562	0.108232
GSDMB	1.004852	0.001576	0.10844
DIAPH1	1.057867	0.001605	0.109222
IL13RA1	1.001496	0.001704	0.111685
TNS4	1.031695	0.001781	0.113891
EMP2	−1.07766	0.001861	0.115604
TXNIP	−1.18813	0.001995	0.118484
LLGL2	1.079593	0.00212	0.120175
ASPH	−1.03794	0.002128	0.120268
QSOX1	1.046049	0.002209	0.122517
NARS	1.01502	0.002219	0.122517
CSNK1E	−1.03495	0.002274	0.123993
TMC5	1.061215	0.002611	0.130769
SF3B1	1.014811	0.002728	0.132083
IGLV2-14	−1.02235	0.002829	0.134358
BRD2	−1.04172	0.002858	0.134601
TUG1	1.078131	0.002916	0.135999
SPINT1	1.007512	0.003412	0.142832
IGHM	−1.19344	0.005126	0.164818
NEAT1	1.102776	0.005743	0.169861
REG4	1.040497	0.01811	0.245883
IGHG1	−1.02939	0.018829	0.248161

## GSEA and GSVA between high- and low-risk groups

**FIGURE 10:**
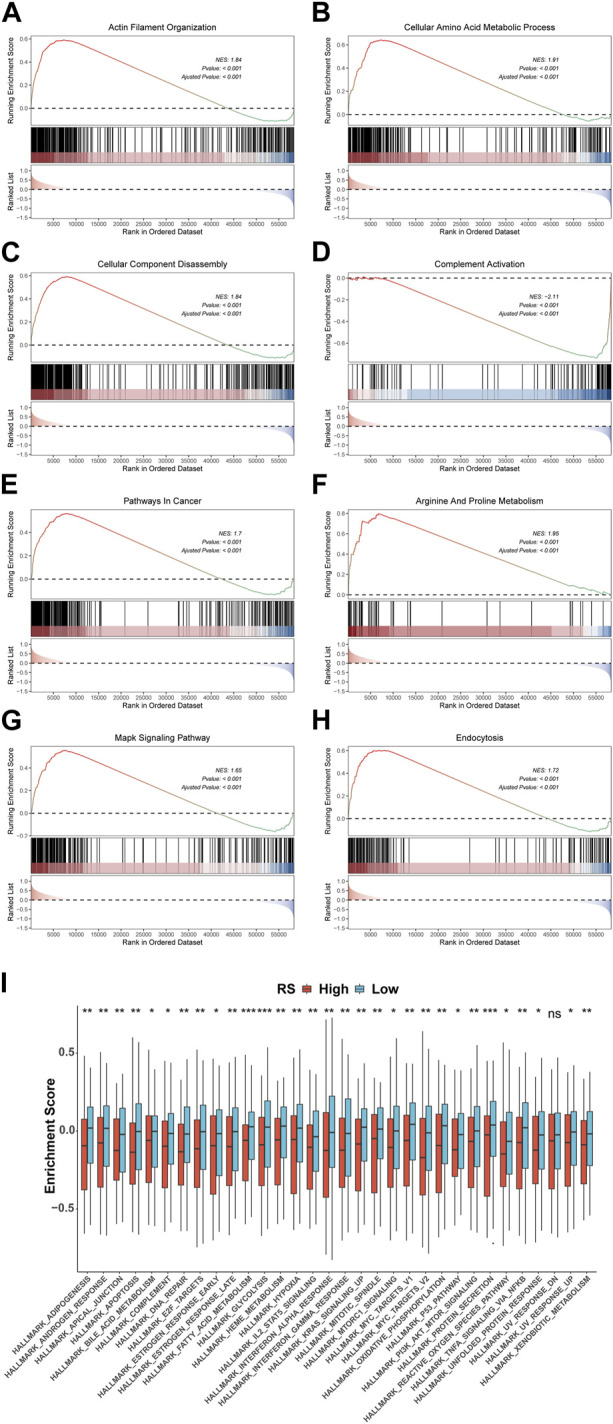
Variations in GO, KEGG, and Hallmark pathway enrichment scores between model groups. GO **(A-D)** and KEGG **(E-H)** enriched the top four pathways with the most significant results based on GSEA. **(I)** Box plot of GSVA of 50 Hallmark pathways. *p < 0.05; **p < 0.01; ***p < 0.001; ****p < 0.0001.

To explore the pathways associated with the RS, we conducted GSEA between 148 high-risk and 148 low-risk groups in TCGA-STAD. The results show that 2404 GO terms and 97 KEGG pathways are significantly enriched. We took the first four pathways of GO and KEGG, respectively, for display, which were GOBP ACTIN FILAMENT ORGANIZATION(Figure 10A), GOBP CELLULAR AMINO ACID METABOLIC PROCESS(Figure 10B), GOBP CELLULAR COMPONENT DISASSEMBLY (Figure 10C), GOBP COMPLEMENT ACTIVATION (Figure 10D) of GO and KEGG PATHWAYS IN CANCER (Figure 10E), KEGG ARGININE AND PROLINE METABOLISM (Figure 10F), KEGG MAPK SIGNALING PATHWAY (Figure 10G), and KEGG ENDOCYTOSIS (Figure 10H) of KEGG. In addition, GSVA results using 50 HALLMARK pathways in the MSigDB database as the reference gene set showed that the enrichment results were significantly different between high- and low-risk groups, except for HALLMARK UV RESPONSE DN (*p* < 0.05) (Figure 10I).

## Discussion

Due to the high incidence of gastric cancer, there is an urgent need to identify effective biomarkers to predict patient prognosis (Sung et al., 2021). The traditional AJCC stage system can roughly predict the clinical survival status of patients, but it cannot effectively distinguish patients under the same stage (Edge and Compton, 2010; Amin et al., 2017). This causes issues in patient treatment, resulting in overtreatment and inadequate treatment. With the development of molecular biology and immunology, there are more and more strategies for the treatment of gastric cancer, and immune checkpoint blockade has become a mainstream treatment (Nagaraju et al., 2021; Lei et al., 2022b; Liabeuf et al., 2022). However, no matter the type of treatment, a reliable biomarker for predicting the prognosis of patients is crucial, and the classification of patients based on biomarkers can effectively facilitate personalized treatment.

Based on this, we integrated prognostic immune-related lncRNAs to construct a prediction model to predict the prognosis of patients and conduct a classification study of patients. The ImmLnc algorithm was applied to identify immune-related lncRNAs. According to the lncRNA expression profile of the TCGA-STAD training dataset, 117 combinations were developed by 10 machine learning algorithms. Further evaluation in two GEO datasets indicated that the ILPM, the optimal model, was the combination of stepwise Cox regression (direction = forward). In terms of predicting OS, the ILPM showed good performance in both TCGA and GEO datasets. In different datasets, the ROC AUC and C-index values were higher in terms of RS, according to the ILPM, compared with other clinical variables (e.g., age, gender, grade, pathologic M stage, pathologic N stage, pathologic T stage, and AJCC stage), which revealed good potential clinical application value. The IPLM enables us to make more accurate predictions and classifications of patients’ prognoses, which is of great significance in clinical application and solves the problem that traditional classification cannot distinguish patients with the same AJCC stage. Compared with the existing prediction models, such as those by Cai et al. (2019), Zheng et al. (2021b), and Lei et al. (2022a), the ILPM continued to perform best.

Afterward, all patients with RS were calculated based on the ILPM, and according to the median of RS, all patients were divided into high- and low-risk groups. Patients in the high-risk group had a worse prognosis and a lower percentage of immune cell infiltration, which is consistent with previous studies showing that cancer cells evade the immune system by having a smaller percentage of immune cells. However, the frequency of mutations was lower in high-risk patients, and in general, the frequency of mutations was higher in patients with poor prognoses, suggesting that our classification is not suitable for studies that predict immunotherapy response based on tumor mutational burden (TMB) (Jardim et al., 2021). On the other hand, it may indicate that gastric cancer patients have different genomic characteristics compared with other cancers and need to be treated differently during immunotherapy.

At the same time, patients in the high-risk group were more sensitive to 18 GDSC drugs (ABT.263, AG.014699, AMG.706, AP.24534, ATRA, axitinib, AZD.0530, BX.795, CCT007093, elesclomol, IPA.3, JNJ.26854165, lenalidomide, methotrexate, Nutlin.3a, OSI.906, PD.0332991, and VX.702). These drugs fight tumors in different ways, for example, ABT.263 (navitoclax), AG.014699 (rucaparib), AMG 706 (motesanib), AP.24534 (ponatinib), AZD.0530 (saracatinib), BX-795, CCT007093, elesclomol, IPA.3, Nutlin-3a (rebemadlin), Osi-906 (linsitinib), Pd-0332991(palbociclib), and Vx-702 were acting as inhibitors of different signaling pathways to fight tumors. In particular, AG.014699 and AZD.0530 were first-generation inhibitors (Creedon and Brunton, 2012; Syed, 2017), and AP.24534 was a second-generation inhibitor (PonatinibLiverTox, 2012). In addition, ATRA, BX-795, CCT007093, elesclomol, and Nutlin-3a were potent inhibitors of pathway molecules. In addition to VX-702, other drugs have therapeutic effects on cancers, such as breast cancer, non-small cell lung cancer, and colon cancer. We found that these drugs may be potential drugs for the treatment of gastric cancer. However, as a drug for the treatment of arthritis (Cohen and Fleischmann, 2010), the application value of VX-702 in gastric cancer still needs further study.

Our analysis of DEG enrichment between the high- and low-risk groups found that DEGs are mainly enriched in certain parts, like the immune response-regulating cell surface receptor signaling pathway, immune response-activating cell surface receptor signaling pathway, immunoglobulin receptor binding, and other immune-related pathways. Descriptions of the differences between the high- and low-risk groups of patients are mainly based on the regulation of immune-related pathways, and these pathways affect the patient’s immune cells and tumor immune microenvironment. High- and low-risk groups of TME verified the results, and the numerous differences between the 50 cancer-related hallmark pathway GSVAs were further highlighted.

In conclusion, based on immune-related lncRNAs, signatures constructed by a variety of machine learning algorithms have effective and stable efficacy in prognostic prediction and classification of patients, the ILPM is an effective and convenient tool for personalized treatment and the clinical diagnosis of GC patients.

## Data Availability

Publicly available datasets were analyzed in this study. These data can be found in a public, open-access repository. The datasets analyzed during the current study are available in the Gene Expression Omnibus (GEO, https://www.ncbi.nlm.nih.gov/geo/) and The Cancer Genome Atlas (TCGA) network (https://cancergenome.nih.gov/).
