# Supplementary figures and images for: Integrating multiple machine learning algorithms for prognostic prediction of gastric cancer based on immune-related lncRNAs
Source: Front Genet. 2023 Apr 4;14:1106724. doi: 10.3389/fgene.2023.1106724 (PMC10111190; doi:10.3389/fgene.2023.1106724)

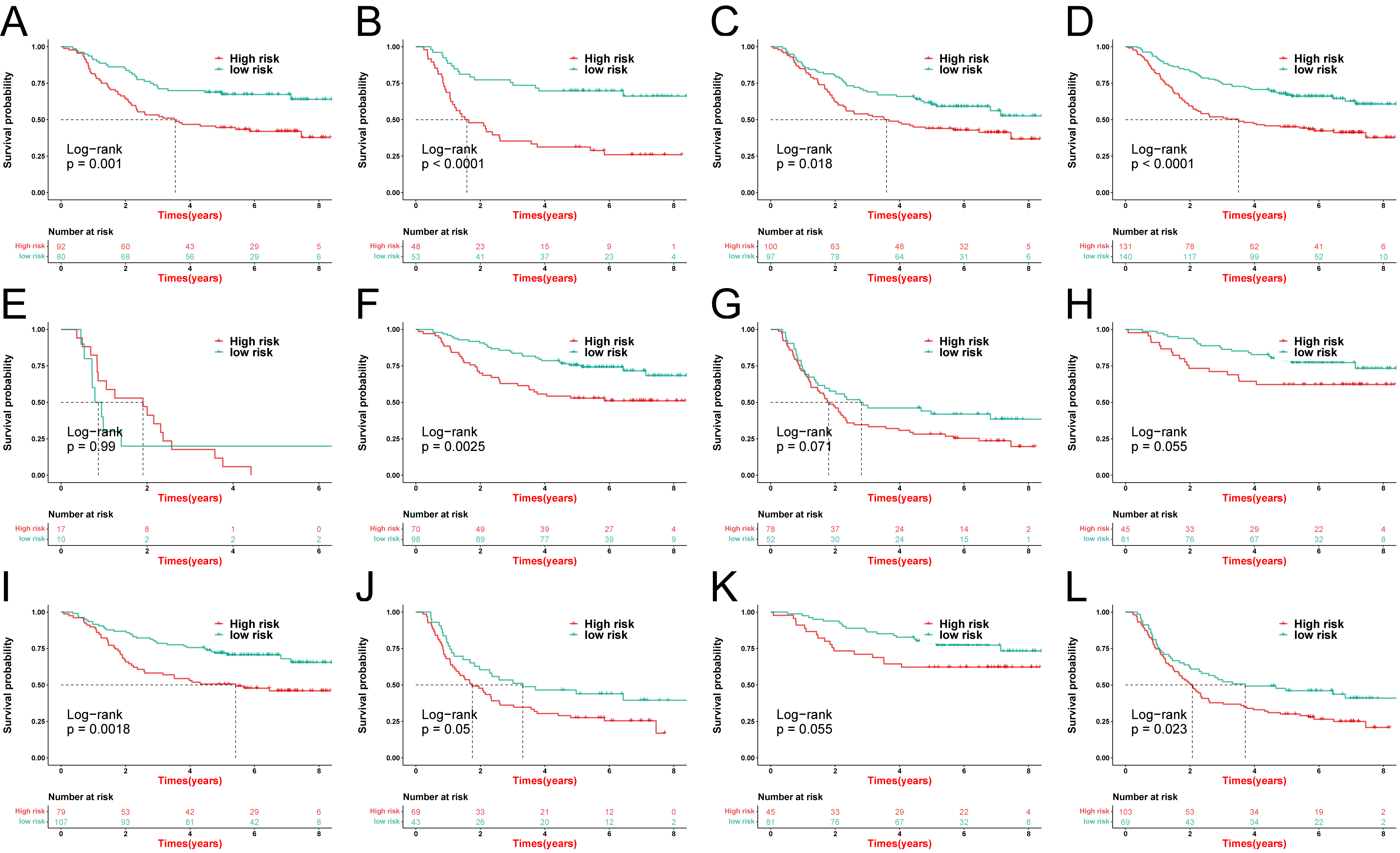

Supplement: Supplementary file 1 [file Image3.TIF]

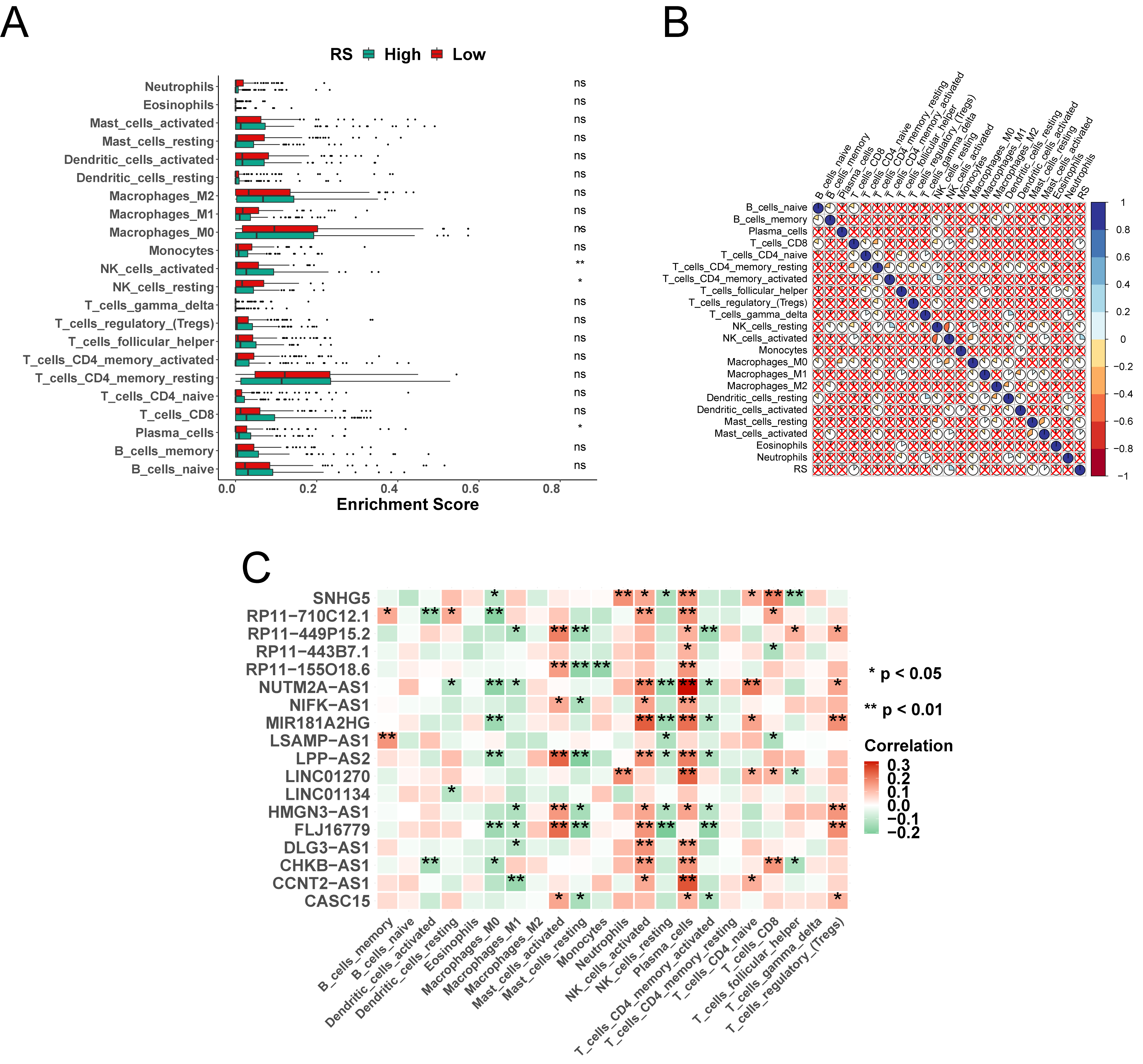

Supplement: Supplementary file 2 [file Image4.TIF]

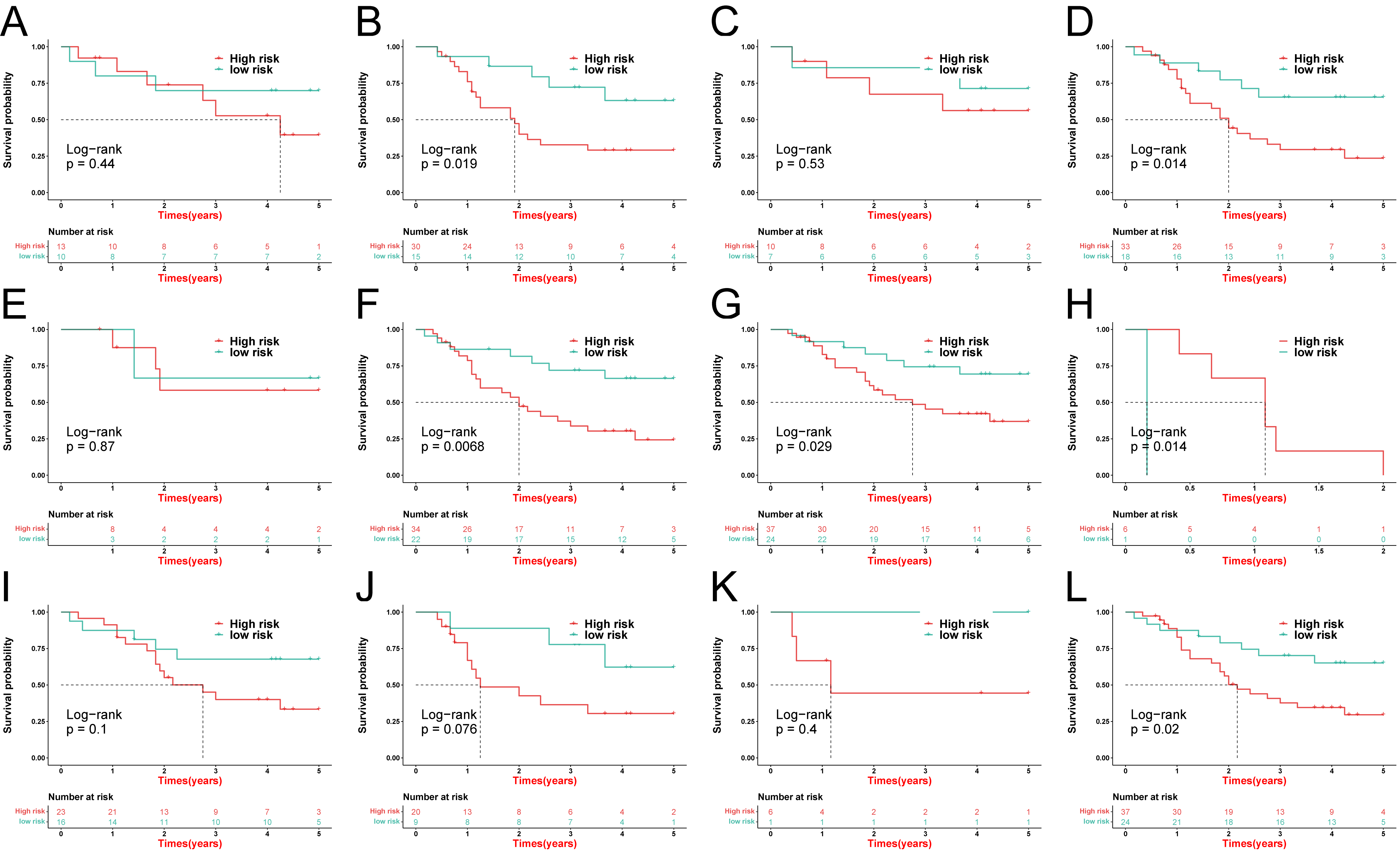

Supplement: Supplementary file 3 [file Image2.TIF]

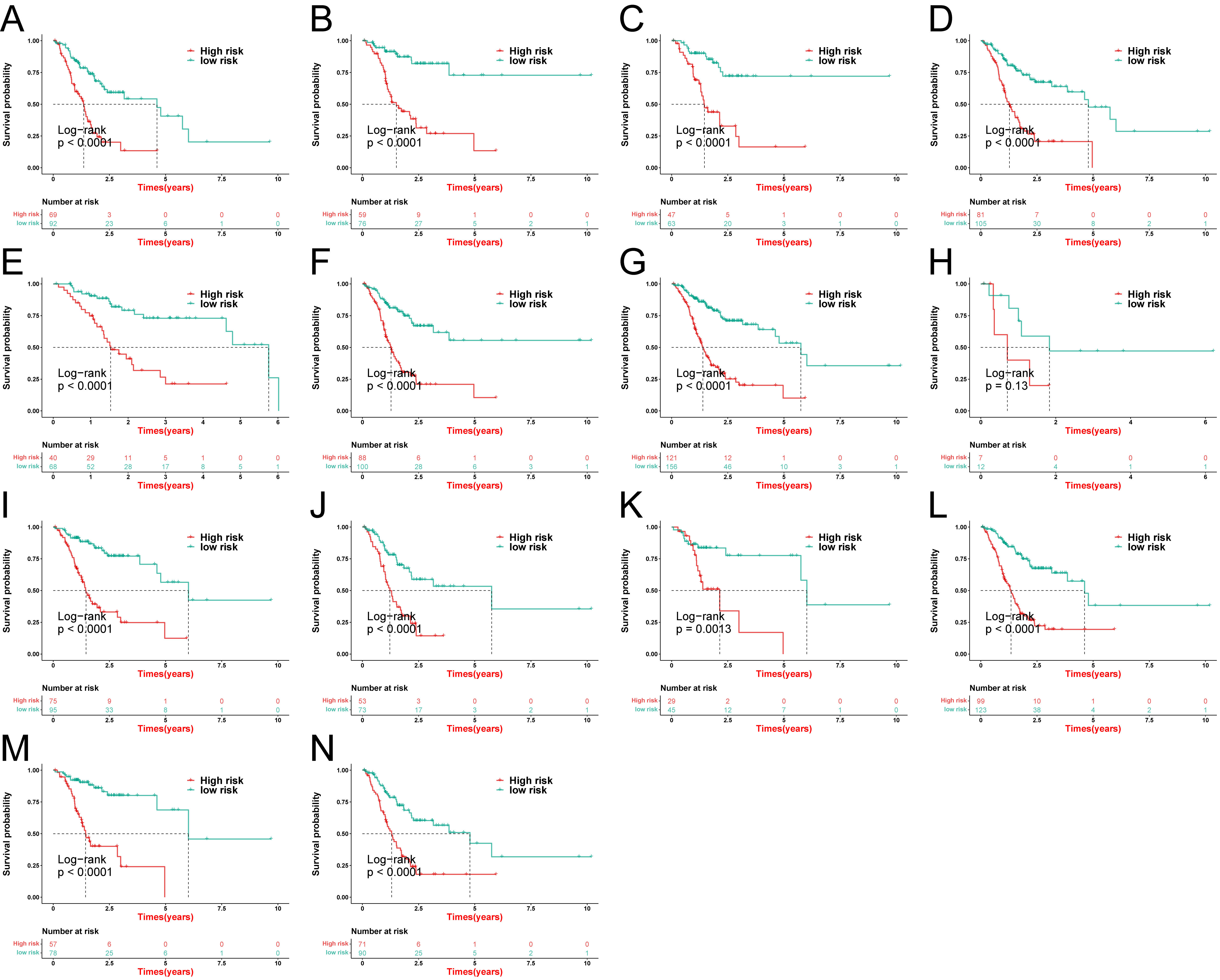

Supplement: Supplementary file 4 [file Image1.TIF]
